# From Rhythm to Relief: Heart Rate Variability as a Window into Anhedonia Response During Agomelatine Treatment in Major Depressive Disorder

**DOI:** 10.3390/medicina61091522

**Published:** 2025-08-25

**Authors:** Chin-Chao Ma, Chu-Ya Yang, Wei-Chou Chang, Alexander T. Sack, Chuan-Chia Chang, Hsin-An Chang

**Affiliations:** 1 Department of Psychiatry, Tri-Service General Hospital Beitou Branch, National Defense Medical University, Taipei 114, Taiwan; touchnature@gmail.com; 2 Department of Psychiatry, Tri-Service General Hospital, National Defense Medical University, Taipei 114, Taiwan; j25176659@gmail.com; 3 Department of Radiology, Tri-Service General Hospital, National Defense Medical University, Taipei 114, Taiwan; weichou.chang@gmail.com; 4 Department of Cognitive Neuroscience, Faculty of Psychology and Neuroscience, Maastricht University, 6211 LH Maastricht, The Netherlands; a.sack@maastrichtuniversity.nl; 5 Department of Psychiatry and Neuropsychology, School for Mental Health and Neuroscience (MHeNs), Brain + Nerve Centre, Maastricht University Medical Centre (MUMC), 6229 HX Maastricht, The Netherlands; 6 Society of Psychophysiology, Taipei 114, Taiwan

**Keywords:** heart rate variability, anhedonia, electrophysiology, major depressive disorder, agomelatine, melatonergic antidepressant, treatment response

## Abstract

*Background and Objectives:* Anhedonia, a core symptom of major depressive disorder (MDD), is a known predictor of treatment response. It has been linked to heart rate variability (HRV), a physiological marker implicated in both MDD and cardiovascular disease. Agomelatine, a melatonergic antidepressant, has shown positive effects on both anhedonia and HRV. But little is known about the relationship between anhedonia improvement and HRV changes. This study aimed to investigate whether early changes in HRV predict anhedonia improvement following 8 weeks of agomelatine monotherapy. *Materials and Methods:* We enrolled 84 unmedicated patients with MDD and 143 age- and sex-matched healthy controls (HCs). Resting-state HRV, indexed by the standard deviation of NN intervals (SDNN), was recorded at baseline for all participants and after 1, 4, and 8 weeks of agomelatine treatment in patients. Anhedonia was assessed using the Snaith–Hamilton Pleasure Scale (SHAPS). *Results:* At baseline, patients exhibited significantly lower SDNN than HCs. After 8 weeks, SDNN levels in patients no longer differed significantly from HCs. SDNN decreased after one week of treatment but increased by week eight. Notably, a smaller reduction in SDNN after one week predicted greater improvement in anhedonia at week eight, filling the gap in the literature needed to facilitate treatment outcome prediction by integrating HRV assessment. *Conclusions:* Here we demonstrate that early reductions in HRV may serve as a predictive biomarker for anhedonia response to agomelatine in MDD. These findings support the potential utility of HRV monitoring to guide personalized treatment strategies.

## 1. Introduction

Anhedonia—a diminished capacity to experience pleasure and reduced responsiveness to rewarding stimuli—is a core symptom of major depressive disorder (MDD). It is frequently observed during major depressive episodes [1,2], may persist as a residual symptom after antidepressant treatment, and has potential as a predictor of treatment response and brain reward system dysfunction [3,4,5]. Anhedonia also poses a challenge for psychopharmacological interventions. For example, first-line antidepressants such as selective serotonin reuptake inhibitors (SSRIs) often demonstrate limited efficacy in alleviating deficits in motivation and reward processing [6,7]. Recent research indicates that agomelatine, an atypical antidepressant acting as a melatonergic receptors (MT1/MT2) agonist and a postsynaptic serotonin receptor 5-HT2c antagonist, can improve anhedonia within one to three weeks of treatment initiation [8,9,10,11]. Further investigation into the neural correlates of anhedonia improvement during agomelatine treatment may help elucidate the underlying neural mechanisms guide the development of novel depression treatments.

Evidence indicates that patients with MDD often display autonomic nervous system (ANS) dysfunction, which may contribute to an increased risk of cardiovascular disease (CVD). The MDD–CVD relationship involves a complex interplay of behavioral, environmental, and biological mechanisms. Indirect contributors include unhealthy behaviors like poor diet, physical inactivity and substance misuse, as well as environmental factors like chronic stress associated with traumatizing experiences during critical developmental stages. Direct biological mechanisms may involve over-stimulation of the hypothalamic–pituitary–adrenal axis (HPA) and heightened immune activity, including elevated levels of pro-inflammatory cytokines [12]. Although the precise mechanisms remain unclear, ANS imbalance has been proposed as a key pathophysiological factor cardiac dysregulation with the onset of depressive symptoms.

Analysis of heart rate variability (HRV) is widely used for measuring the ANS function and understanding associations between the ANS and affective states/disorders [13]. HRV refers to the complex beat-to-beat variation in heart rate produced by the interplay of sympathetic and parasympathetic neural activity at the sinus node of the heart. Low HRV has been shown to predict adverse cardiovascular events even in individuals without prior CVD [14]. MDD encompasses diverse symptom domains, and certain subtypes are particularly associated with low HRV. For example, melancholic MDD is linked to profoundly reduced HRV [15], and some evidence suggests that the MDD–HRV association reported in the literature may be driven primarily by melancholic features [16]. In the Diagnostic and Statistical Manual of Mental Disorders, Fifth Edition (DSM-5), melancholic features are defined as a clinical subtype of MDD characterized by affect disturbances (anhedonia and/or nonreactive mood), psychomotor disturbance, cognitive impairment, and vegetative dysfunction. Animal studies have shown that stressor-induced behavioral indicators of anhedonia is associated with decreased HRV and sympathovagal imbalance, suggesting that among diverse depressive symptomatology, anhedonia may the strongest translational potential for studying HRV and depressive pathophysiology [17]. In adolescents, anhedonia has been found to be the depressive symptom most strongly and consistently linked to HRV variance over time [18], and a study in healthy adults also indicates an association between reduced HRV and anhedonic behaviors [19]. Together, these findings highlight HRV as a promising biomarker for exploring the neural mechanisms underlying anhedonia improvement during antidepressant treatment.

Different classes of antidepressants exert distinct effects on HRV in MDD patients [20]. A recent meta-analysis of randomized placebo-controlled trials (RCTs) and pre–post studies found that SSRIs significantly reduced HRV in RCTs, while tricyclic antidepressants (TCAs) significantly decreased HRV in pre–post studies [21]. In contrast, both agomelatine and SSRIs were associated with HRV increases in pre–post studies. Our previous work demonstrated that HRV is reduced in MDD patients compared to healthy controls [22] and 6-week agomelatine monotherapy is associated with a significant increase in HRV [23,24]. Compared with paroxetine, a SSRI, agomelatine has a stronger HRV-enhancing effect, potentially due to its melatonergic agonism and 5-HT2c antagonism on autonomic regulation [23]. However, it remains unknown whether increases or dynamic early changes in HRV are associated with anhedonia improvement following agomelatine monotherapy. To address this gap, the present study was designed with two objectives. The primary aim was to examine whether early changes in HRV after 1-week agomelatine treatment predict anhedonia improvement at the end of treatment. The second was to compare the differences in HRV between unmedicated patients with MDD and healthy controls (HCs) and between MDD patients following 8-week agomelatine monotherapy and HCs.

## 2. Materials and Methods

### 2.1. Participants

Eighty-four unmedicated individuals diagnosed with DSM-5-defined MDD were recruited for a prospective, observational, single-center cohort study where eligible patients received eight weeks of agomelatine monotherapy (25 mg/day). The study was approved by the Ethics Committees of the Tri-Service General Hospital (No. of IRB: B202205019), and written informed consent was obtained from all participants. The current sample overlaps with that reported in our previous EEG and functional near-infrared spectroscopy (fNIRS) studies, in which design, methodology, and recruitment procedures are detailed elsewhere [25,26,27]. Relevant aspects for the present study are summarized here. Participants had not received any antidepressant medication within two weeks prior to baseline neurophysiological assessment (i.e., they were either drug-naïve or had discontinued antidepressants). The inclusion criteria are: (1) patients aged between 20 and 65 years, (2) being able to consent to participate in the study, (3) recently having a major depressive episode with low suicide risk as assessed by the Mini International Neuropsychiatric Interview (MINI) [28], and (4) a Montgomery Åsberg Depression Rating Scale (MADRS) score > 21 on the screening day. The exclusion criteria for this study are as follows: (1) individuals with specific medical conditions, including hepatic failure (such as cirrhosis or active liver disease), any form of transaminase abnormalities, heart failure, sepsis, renal impairment, unstable hypertension, hypotension, and diabetes mellitus with poor glycemic control; (2) individuals with a history of bipolar disorder or schizophrenia spectrum disorders; (3) individuals with an active substance use disorder, excluding caffeine and tobacco; (4) individuals diagnosed with major neurocognitive disorders, sensory impairments, epilepsy, or brain injuries; (5) individuals who are pregnant or breastfeeding; (6) individuals concurrently using potent CYP1A2 isoenzyme inhibitors (e.g., fluvoxamine, ciprofloxacin); and (7) individuals exhibiting hypersensitivity to agomelatine or its excipients. MDD participants were rated by experienced psychiatrists with the MADRS [29] and the Hamilton Anxiety Rating Scale (HARS). Anhedonia was assessed using the Chinese version of Snaith–Hamilton Pleasure Scale (SHAPS) [30,31]. SHAPS is a 14-item self-report questionnaire to assess the subject’s pleasure experience in the “last few days”. It is rated from definitely agree to definitely disagree on a 4-point Likert scale (i.e., scoring from 1 to 4) and the total score ranges from 14 to 56. High scores indicate more severe anhedonia. The self-reported overall severity of depression and anxiety was assessed using the Patient Health Questionnaire (PHQ-9) [32], and Generalized Anxiety Disorder 7 (GAD-7) [33], respectively. The abovementioned clinical outcomes were measured at baseline, at weeks 1, 4, and 8 after agomelatine treatment.

The normal control (HC) group included 143 healthy participants. Recruitment and exclusion processes have been described elsewhere [26,34]. In summary, all healthy participants underwent a comprehensive medical evaluation, which encompassed biochemical analyses, blood pressure assessments, electrocardiograms, physical examinations, and thoracic radiographs. None of the participants exhibited any organic diseases, including but not limited to renal or hepatic disorders, cardiovascular diseases, metabolic conditions, neurological disorders, malignancies, or obesity. The participants were determined to be free of mental disorders according to evaluations conducted with the Chinese Version of the MINI [28] by a trained research assistant. Furthermore, it was established through self-reporting that none of the participants had been on any medications for a minimum of one month prior to their enrollment in the study. The severity of depression and anxiety among the healthy subjects was evaluated using the BDI-II and the BAI.

### 2.2. Heart Rate Variability (HRV) Measurement

The detailed procedures were reported in our previous studies [23,24,35]. Briefly, the participants first sat quietly for 20 min, then a lead I electrocardiogram was recorded for 5 min. To control for fluctuations in the autonomic nervous system over the course of a day, all electrocardiogram recordings were carried out in a quiet and air-conditioned room between 9 and 11 a.m. HC participants were measured only once whereas MDD participants were measured at baseline, at weeks 1, 4, and 8 after agomelatine treatment. The payment was offered to the subjects to reimburse them for the transportation expense of each visit. An HRV analyzer acquired, stored, and processed the electrocardiography signals. Our computer algorithm then identified each QRS complex and rejected each ventricular premature complex or noise according to its likelihood in a standard QRS template. Signals were recorded at a sampling rate of 512 Hz, using an 8-bit analog-to-digital converter. Stationary R-R interval values were re-sampled and interpolated at a rate of 7.11 Hz to produce continuity in the time domain. The standard deviation of NN intervals (SDNN), a time-domain measure of HRV, measures the standard deviation of the time between normal heartbeats (NN intervals) over 5 min. SDNN is calculated by taking the square root of the variance in NN intervals, reflecting all cyclic components responsible for variability during the recording period. It is considered an estimate of overall HRV and the “gold standard” for medical stratification of cardiac risk. A non-parametric fast Fourier transformation was used to perform power spectral analysis. The direct current component was deleted, and a Hamming window was used to attenuate the leakage effect. The power spectrum was subsequently quantified into standard frequency-domain measurements, namely low-frequency power (LF, 0.04–0.15 Hz), high-frequency power (HF, 0.15–0.40 Hz), and the ratio of LF to HF power (LF/HF). Vagal control of HRV is represented by HF, whereas both vagal and sympathetic control of HRV are jointly represented by LF. The LF/HF ratio could mirror sympatho-vagal balance or sympathetic modulation, with a larger LF/HF ratio indicating a greater predominance of sympathetic activity over cardiac vagal control.

### 2.3. Statistics

Statistical analyses were conducted utilizing IBM SPSS Statistics 24.0 software (IBM SPSS Inc., Chicago, IL, USA). All data were checked for deviations from a Gaussian distribution. Because the study had less than 3% missing data, complete case analyses were used. For the comparisons of continuous variables between the pre-treatment MDD group and the healthy control (HC) group and between the post-treatment MDD group and the HC group, independent *t*-tests were used for parametric variables and the Mann–Whitney U tests for nonparametric variables. The χ^2^ tests were used to examine between-group differences in discrete variables. For the comparisons of continuous variables between baseline and each post-baseline visit, paired *t*-tests were used for parametric variables and the Wilcoxon Signed-Rank tests for nonparametric variables. Spearman’s correlation analysis was used to investigate the relationship between the change in SDNN from baseline to 1 week after treatment and the change in SHAPS score from baseline to 8 weeks after treatment. Statistical significance was established at *p* < 0.05 (two-tailed) for the primary outcomes (i.e., the correlation between the change in SDNN one week after treatment and the change in SHAPS score 8 weeks after treatment). To mitigate the increased risk of Type 1 error associated with multiple statistical tests for other secondary outcomes, Bonferroni corrections were applied. Cohen’s d effect sizes and power analysis were calculated by using G*power Version 3.1.9.4.

## 3. Results

### 3.1. Sample Characteristics

**Figure 1:**
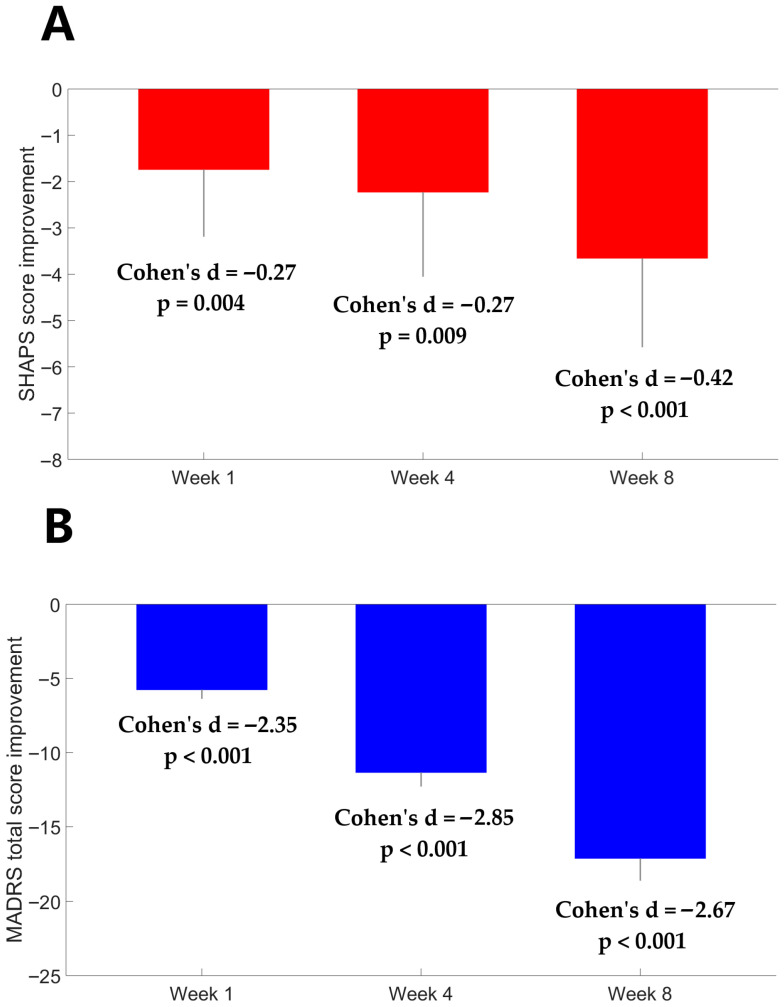
The average improvement in (**A**) SHAPS and (**B**) MADRS scores 1 week, 4 weeks, and 8 weeks after agomelatine monotherapy. Error bars represent 95% confidence intervals for the mean. The SHAPS and MADRS scores at baseline versus at each post-baseline assessment were compared using Wilcoxon signed-rank tests.

Table 1 presents a summary of the demographic and clinical characteristics of the study participants. Among the 84 patients, 35 (41.67%) were antidepressant drug-naïve and 49 (58.33%) were not taking any antidepressant for at least more than 2 months. There were no significant differences in age, sex, body mass index, exercise levels or smoking status between patients with MDD and HCs (all *p* values > 0.05). As can be seen in Figure 1, there was a significant improvement in anhedonia one week after agomelatine monotherapy (Z = −2.86, Cohen’s d = −0.27, *p* = 0.004). The improvement remained 4 weeks (Z = −2.62, Cohen’s d = −0.27, *p* = 0.009) and 8 weeks (Z = −3.9, Cohen’s d = −0.42, *p* < 0.001) after treatment. Depression severity improvement was observed one week (Z = −7.82, Cohen’s d = −2.35, *p* < 0.001), 4 weeks (Z = −7.92, Cohen’s d = −2.85, *p* < 0.001) and 8 weeks after treatment (Z = −7.92, Cohen’s d = −2.67, *p* < 0.001).

### 3.2. HRV Metrics Between the Pre-Treatment MDD Cohort and the HC Group

**Figure 2:**
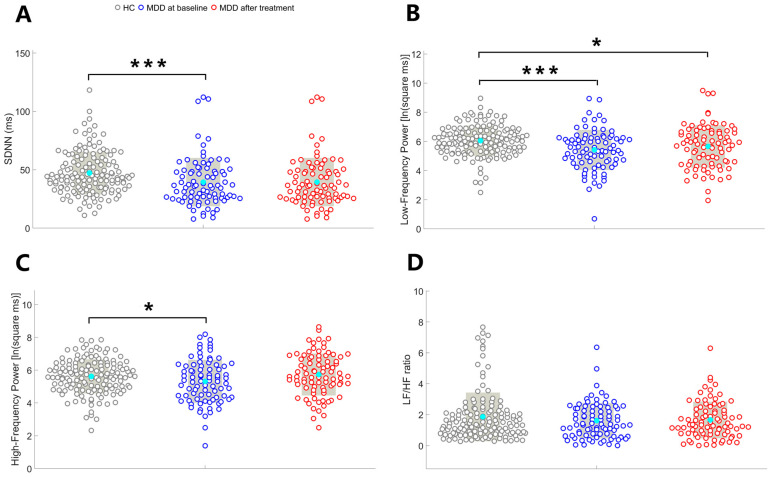
Comparisons of HRV metrics including SDNN (**A**), LF (**B**), HF (**C**), and LH/HF ratio (**D**) between pre-treatment patients with major depressive disorder (MDD) and healthy controls (HCs) as well as between post-treatment patients with MDD and HCs. Each circle represents the value of HRV metrics of each participant. The cyan dot indicates the mean value of the HRV metrics. The gray box indicates standard deviations. * *p* < 0.05; ** *p* < 0.01; *** *p* < 0.001.

As illustrated in Figure 2, SDNN (U = −3.63, *p* < 0.001) and LF (t = −3.66, *p* < 0.001) were significantly lower in pre-treatment patients with MDD compared to HCs. HF was lower in pre-treatment patients with MDD compared to HCs (t = −2.07, *p* = 0.04); however, this difference did not achieve the Bonferroni-adjusted threshold for significance. There was no between-group difference in the LF/HF ratio (t = −0.96, *p* = 0.34).

### 3.3. HRV Metrics from the Baseline to the End of the Treatment

**Figure 3:**
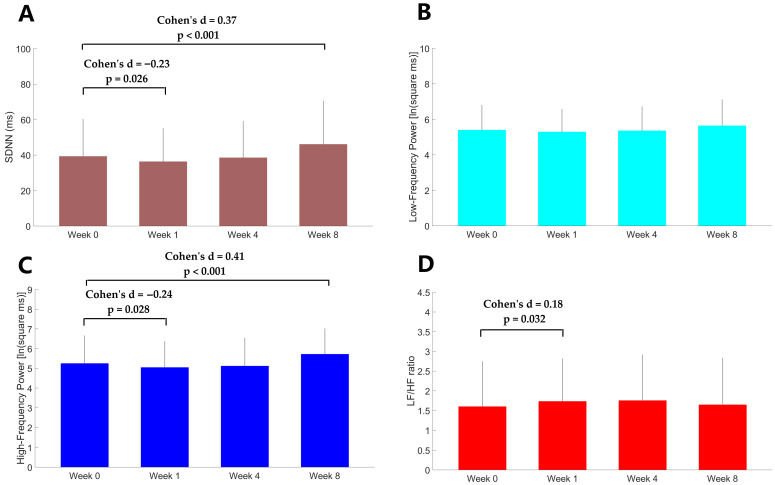
The values of HRV metrics including SDNN (**A**), LF (**B**), HF (**C**), and LH/HF ratio (**D**) at baseline (week 0), 1 week, 4 weeks, and 8 weeks after agomelatine monotherapy. Error bars represent standard deviations for the mean. The SDNN and LH/HF ratio at baseline versus at each post-baseline assessment were compared using Wilcoxon signed-rank tests. LF and HF were compared using paired *t* tests.

Compared to baseline, there was a significant reduction in SDNN (Z = −7.82, Cohen’s d = −0.23, *p* = 0.026) one week after treatment (Figure 3). HF (t = −2.24, Cohen’s d = −0.24, *p* = 0.028) was reduced and LF/HF ratio (Z = 2.15, Cohen’s d = 0.18, *p* = 0.032) was increased from baseline to one week after treatment but this differences did not achieve the Bonferroni-adjusted threshold for significance. SDNN (Z = 3.60, Cohen’s d = 0.37, *p* < 0.001) and HF (t = 3.8, Cohen’s d = 0.41, *p* < 0.001) were significantly increased 8 weeks after treatment. Other results were all nonsignificant.

### 3.4. HRV Metrics Between the Post-Treatment MDD Cohort and the HC Group

As illustrated in Figure 2, no between-group differences were identified in the SDNN (U = −1.07, *p* = 0.29), HF (t = −0.69, *p* = 0.49), and LF/HF ratio (t = −0.84, *p* = 0.40). LF was lower in post-treatment patients with MDD compared to HCs (t = −2.18, *p* = 0.03); however, this difference did not achieve the Bonferroni-adjusted threshold for significance.

### 3.5. Correlation Analyses

**Figure 4:**
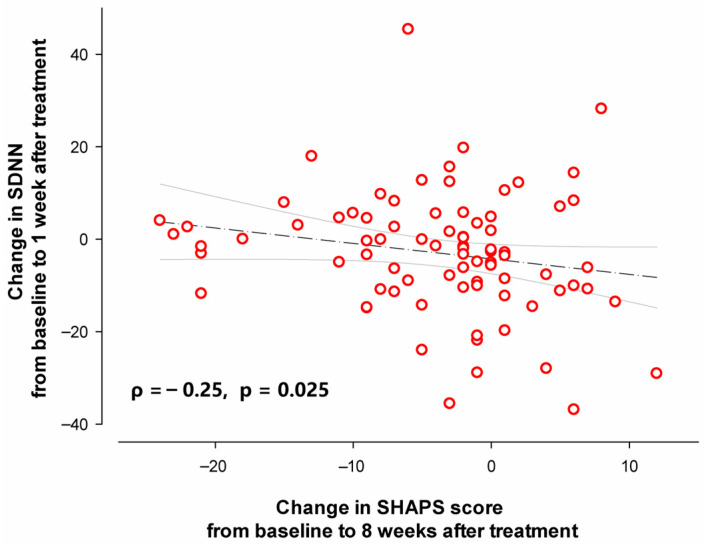
The correlation between the change in SDNN from baseline to 1 week after treatment and the change in SHAPS score from baseline to 8 weeks after treatment. Each red circle represents a single participant with major depressive disorder. The regression line and 95% confidence intervals for the linear regression slope are shown.

Correlation analysis showed that the change in SDNN from baseline to 1 week after treatment exhibited a negative correlation with the change in SHAPS score from baseline to 8 weeks after treatment (ρ = −0.25, *p* = 0.025, Figure 4). Specifically, less reduction in SDNN at 1 week after treatment predicted greater anhedonia improvement following 8-week treatment with agomelatine. The present total sample had a power of 0.753 to detect the correlation as the primary outcome of the study.

### 3.6. Summaries of the Main Results

MDD patients at baseline exhibited significantly lower SDNN than HCs. After 8 weeks, SDNN levels in patients did not differ significantly from HCs. SDNN decreased after one week of treatment but increased by week eight. A smaller reduction in SDNN after one week predicted greater anhedonia improvement at week eight.

## 4. Discussion

In line with our primary hypothesis, we found that early HRV changes at one week after treatment onset were associated with anhedonia improvement following eight weeks of agomelatine monotherapy. We also observed that unmedicated patients with MDD exhibited significantly lower HRV (particularly LF-HRV) as compared to HCs. The difference was no longer statistically significant following eight weeks of agomelatine monotherapy.

Consistent with recent research [8,9,10,11], significant improvement in anhedonia was evident soon after initiating agomelatine monotherapy and persisted throughout the eight-week treatment period. The mechanism by which anhedonia improves with agomelatine treatment remains incompletely understood, though several possibilities have been proposed. Agomelatine is known for its unique mode of action of synergy between melatonergic receptor activation and 5-HT2c receptor antagonism [36]. It can dose-dependently suppress neuronal firing activity in the suprachiasmatic nucleus—the center controlling circadian rhythms, including wake–sleep, and autonomic system rhythms. In animal models, agomelatine has been shown to reverse anhedonia-like deficits induced by chronic constant light exposure by restoring plasma melatonin levels and circadian patterns [37]. In depressed patients, agomelatine shifted the dim light melatonin onset (DLMO)—the most accurate marker for circadian pacemaker assessment—3.6 h earlier, suggesting a strong link between depression improvement and circadian phase adjustment [38]. Antagonizing 5-HT2C receptors may be another strategy for treating anhedonia. For example, treatment with agomelatine or SB242084 (an antagonist of 5-HT2C receptors) can reverse the anhedonia-like behaviors in mice with knockout of the subunit 2D of the N-methyl-d-aspartate (NMDA) glutamate ionotropic receptor [39]. In addition, anhedonia in MDD has been linked to functional abnormalities in the reward circuit [40], a neural network enriched with dopaminergic neurons [41]. Growing evidence also connects anhedonia with disrupted melatonin circadian rhythms in MDD. For example, a recent cross-sectional study in MDD patients showed that the peak phase of melatonin circadian rhythms negatively correlated with anhedonia severity [42]. In depressed mice, melatonin treatment increased the expression of dopamine-related proteins [43]. These findings suggest that normalizing circadian rhythms—such as through melatonin-based interventions—may improve anhedonia by influencing the dopaminergic system. Agomelatine’s ability to indirectly modulate dopaminergic activity via melatonin rhythm regulation and neocortical 5-HT2c receptor antagonism likely plays a key role in its rapid and robust effects on anhedonia.

Our study showed that HRV, reduced in unmedicated MDD patients, significantly increased after eight weeks of agomelatine monotherapy. This improvement was mainly due to increased parasympathetic activity, as indexed by HF-HRV. These results align with prior studies showing that HRV is lower in MDD than in healthy controls [44] and that parasympathetic activity is increased after agomelatine monotherapy for at least six weeks [23,24]. Moreover, in our sample, HRV nearly returned to normal levels after agomelatine treatment—an outcome rarely seen with other antidepressants [20,45,46]. This effect may be attributable to agomelatine’s combined melatonergic receptor agonism and 5-HT2c receptor antagonism, which can increase cardiac vagal activity and reduce sympathetic tone. Supporting evidence includes findings that a single 2 mg melatonin dose increased vagal tone and decreased sympathetic tone in healthy men [47], and animal studies showing that chronic melatonin reduces heart rate via sympathetic inhibition [48]. Additionally, 5-HT2c receptor activation in the nucleus tractus solitarius inhibits cardiac vagal activity, while antagonism prevents this inhibition [49,50]. Notably, research has found no significant HRV differences between remitted MDD patients and HCs [51], underscoring the need for caution when attributing observed HRV changes solely to agomelatine.

Contrary to our expectations, early HRV changes at one week were characterized by a significant reduction, possibly due to a trend-level decrease in parasympathetic tone (HF-HRV) and a trend-level increase in sympathetic modulation (LF/HF ratio). Disrupted circadian rhythms—including changes in melatonin levels and core body temperature—are known to contribute to MDD pathophysiology, and HRV serves as a useful circadian rhythm proxy [36,52]. Drug-free MDD patients have been shown to reach peak HRV oscillation (SDNN) in the morning (~06:00) and trough in the evening (~18:00) [52]. Agomelatine is known to phase-advance circadian changes in melatonin, core body temperature, and the timing of daily heart rate decreases [36]. Repeated melatonin receptor activation can phase-advance HRV indices by shifting their endogenous circadian timing [53]. Given that it typically takes at least six weeks of 25 mg/day agomelatine to significantly and stably increase HRV [23,24], this early phase-advancing effect may explain the initial HRV reduction. Ultimately, circadian normalization by agomelatine may benefit dopaminergic function in the reward circuit, contributing to both short- and long-term improvements in anhedonia.

Earlier research suggested that HRV could serve as a biomarker for predicting responses to serotonergic antidepressants [54]. Our study is the first to show that early HRV changes can predict anhedonia improvement with a melatonergic antidepressant. HRV, particularly vagal modulation, is a valuable indicator of circadian disruption in MDD [15,16,18]. While it might seem counterintuitive that a smaller reduction in HRV after one week predicted greater anhedonia improvement at eight weeks, this may reflect a subset of patients who experienced an early, mild HRV increase due to agomelatine’s vagotonic effects [23,24], partially offsetting its phase-advancing effect. Both processes likely contributed to improved anhedonia. Taken together, our findings suggest that early HRV dynamics could be a clinically relevant biomarker for predicting anhedonia improvement in MDD patients receiving agomelatine. Further studies are needed to explore the neurobiological links between circadian rhythm modulation in HRV and anhedonia, and to confirm its predictive value.

The present study has several significant limitations. First, it was a prospective observational cohort study that analyzed only patients with complete HRV and clinical data, introducing possible selection bias. It needs to take extra caution when making causal interpretations for agomelatine-related HRV and anhedonia changes, as these may reflect a broader therapeutic process involving improvements in both autonomic and depressive symptoms. A recent meta-analysis found that SSRIs have mixed effects on HRV, decreasing it in RCTs but increasing it in pre–post studies [21]. Future placebo-controlled RCTs are necessary to validate our findings. Second, in the absence of predictive modeling, sensitivity/specificity analysis, or control for confounding factors, the designation of HRV as a predictive biomarker may be premature and should be viewed as preliminary and exploratory, calling for rigorous validation in future studies. Finally, short-term HRV changes should be considered tentative, as they may be influenced by agomelatine’s phase-advancing effects. Future work should employ continuous 24 h Holter ECG monitoring and circadian rhythm analyses to better characterize these effects.

## 5. Conclusions

Despite these limitations, our findings demonstrate that HRV is reduced in unmedicated MDD patients compared to healthy controls, and that both HRV and anhedonia significantly improve after eight weeks of agomelatine monotherapy. Aligning with previous research, intra-individual pre–post comparisons in our study showed that anhedonia symptoms in MDD patients improve by the first week of agomelatine treatment. Early HRV changes at one week predicted more anhedonia improvement following eight weeks of agomelatine monotherapy. Our study supports the potential utility of 5 min HRV monitoring to guide personalized treatment strategies in MDD, with minimal patient burden. These findings warrant further validation in placebo-controlled RCTs.

## Figures and Tables

**Table 1:** Demographics, clinical characteristics, and symptomatology of the participants.

Variables	Pre-Treatment MDD	Post-Treament MDD	Healthy Controls	t/Z or χ^2^	*p*-Value
Numbers	84	84	143		
Age, years, mean (SD)	37.42 ± 12.04	-	34.97 ± 8.24	1.36	0.17 ^a^
Females, n (%)	51 (60.71)	-	79 (55.24)	0.65	0.42 ^a^
Education, years, mean (SD)	15.55 ± 1.20	-	15.94 ± 1.30	−1.64	0.10 ^a^
BMI	23.40 ± 5.05	-	23.81 ± 3.73	0.64	0.52 ^a^
Weekly regular exercise, hours	0.69 ± 0.71	-	0.75 ± 0.73	0.58	0.56 ^a^
Current smoker, N (%)	10 (11.90)	-	12 (8.39)	0.75	0.39 ^a^
SHAPS	29.45 ± 7.95	25.79 ± 8.56	-	3.87	**<0.001** ^b^
MADRS	33.85 ± 4.98	16.68 ± 5.72	-	24.49	**<0.001** ^b^
HARS	22.07 ± 6.18	6.70 ± 4.47	-	19.81	**<0.001** ^b^
PHQ-9	16.90 ± 5.64	11.86 ± 6.88	-	7.93	**<0.001** ^b^
GAD-7	14.04 ± 5.13	10.39 ± 5.79	-	7.35	**<0.001** ^b^
BDI	-	-	6.46 ± 5.60	-	-
BAI	-	-	3.82 ± 4.23	-	-

Abbreviations: SD, standard deviation; BMI, body mass index (calculated as weight in kilograms divided by height in meters squared); BDI, Beck Depression Inventory-II; BAI, Beck Anxiety Inventory; MADRS, Montgomery Åsberg Depression Rating Scale; HARS, Hamilton Anxiety Rating Scale; PHQ-9, Patient Health Questionnaire; GAD-7, Generalized Anxiety Disorder 7-item; SHAPS, Snaith-Hamilton Pleasure Scale. Notes: *p* values in bold represent statistical significance. ^a^ *p* values for pre-treatment MDD patients versus healthy controls; ^b^ *p* values for pre-treatment MDD patients versus post-treatment MDD patients.

## Data Availability

The original contributions presented in this study are included in the article. Further inquiries can be directed to the corresponding authors.
